# Nomogram model based on preoperative serum thyroglobulin and clinical characteristics of papillary thyroid carcinoma to predict cervical lymph node metastasis

**DOI:** 10.3389/fendo.2022.937049

**Published:** 2022-07-15

**Authors:** Qungang Chang, Jieming Zhang, Yaqian Wang, Hongqiang Li, Xin Du, Daohong Zuo, Detao Yin

**Affiliations:** ^1^ Department of Thyroid Surgery, The First Affiliated Hospital of Zhengzhou University, Zhengzhou, China; ^2^ Key Medicine Laboratory of Thyroid Cancer of Henan Province, Zhengzhou, China; ^3^ Department of Oncology, The First Affiliated Hospital of Zhengzhou University, Zhengzhou, China; ^4^ Department of Surgery, The First Affiliated Hospital of ZhengZhou University, Zhengzhou, China; ^5^ Engineering Research Center of Multidisciplinary Diagnosis and Treatment of Thyroid Cancer of Henan Province, Zhengzhou, China

**Keywords:** nomogram, lymph node metastasis, preoperative serum thyroglobulin, papillary thyroid carcinoma, surgery

## Abstract

**Objective:**

Preoperative evaluation of cervical lymph node metastasis (LNM) in papillary thyroid carcinoma (PTC) has been one of the serious clinical challenges. The present study aims at understanding the relationship between preoperative serum thyroglobulin (PS-Tg) and LNM and intends to establish nomogram models to predict cervical LNM.

**Methods:**

The data of 1,324 PTC patients were retrospectively collected and randomly divided into training cohort (n = 993) and validation cohort (n = 331). Univariate and multivariate logistic regression analyses were performed to determine the risk factors of central lymph node metastasis (CLNM) and lateral lymph node metastasis (LLNM). The nomogram models were constructed and further evaluated by 1,000 resampling bootstrap analyses. The receiver operating characteristic curve (ROC curve), calibration curve, and decision curve analysis (DCA) of the nomogram models were carried out for the training, validation, and external validation cohorts.

**Results:**

Analyses revealed that age, male, maximum tumor size >1 cm, PS-Tg ≥31.650 ng/ml, extrathyroidal extension (ETE), and multifocality were the significant risk factors for CLNM in PTC patients. Similarly, such factors as maximum tumor size >1 cm, PS-Tg ≥30.175 ng/ml, CLNM positive, ETE, and multifocality were significantly related to LLNM. Two nomogram models predicting the risk of CLNM and LLNM were established with a favorable C-index of 0.801 and 0.911, respectively. Both nomogram models demonstrated good calibration and clinical benefits in the training and validation cohorts.

**Conclusion:**

PS-Tg level is an independent risk factor for both CLNM and LLNM. The nomogram based on PS-Tg and other clinical characteristics are effective for predicting cervical LNM in PTC patients.

## Introduction

Thyroid cancer has been one of the most common and gradually increasing endocrine malignancies in recent years. At approximately 90%, papillary thyroid carcinoma (PTC) has been the most common pathological type (1). It is typically inert and usually shows excellent prognosis to standardized comprehensive treatments (including surgical resection, iodine ablation, and TSH inhibition therapies) (2). However, such clinical features of PTC as the large size of tumor, extrathyroidal extension (ETE), multifocality, lymph node metastasis (LNM), and distant metastasis (3–6) still lead to a poor prognosis. As a distinctive risk factor for distant metastasis and postoperative local recurrence (7, 8), LNM is prone to early PTC (9, 10). Moreover, it can significantly and negatively impact the survival rate and quality of life of the patients (11, 12). Thus, a reasonable and standardized surgical scope (especially lymph node dissection) is essential for its prognosis.

Currently, imaging examinations (mainly B ultrasound and computed tomography) and cytology pathology are the main means of preoperative evaluation of cervical lymph nodes in PTC patients. However, the sensitivity of the methods is mostly below 70% (13, 14). Accordingly, these preoperative examinations may not provide sufficient evidence for surgical decisions in many cases. Therefore, a preoperative evaluation of cervical LNM has remained one of the important clinical challenges.

As a kind of large glycoprotein, thyroglobulin (Tg) is the substrate of thyroid hormone synthesis (15). Tg can be produced by normal thyroid follicular epithelial cells and well-differentiated malignant thyroid tumor cells (16). Currently, serum Tg is mainly used as one of the indicators for monitoring the recurrence of patients with differentiated thyroid cancers (DTC) (17). In addition, fine needle aspiration washout thyroglobulin (FNA-Tg) is used for the diagnosis of cervical LNM (18). However, few studies have investigated the correlation between PS-Tg and cervical LNM in PTC patients. Previous studies revealed that PS-Tg is related to tumor burden and may help to predict LNM (19, 20). In the present study, the relationship between PS-Tg and cervical LNM in PTC patients is retrospectively reviewed. Furthermore, nomogram models are developed by combining PS-Tg with other clinical characteristics to predict CLNM and LLNM, respectively.

## Materials and methods

### Study patients

**Figure 1:**
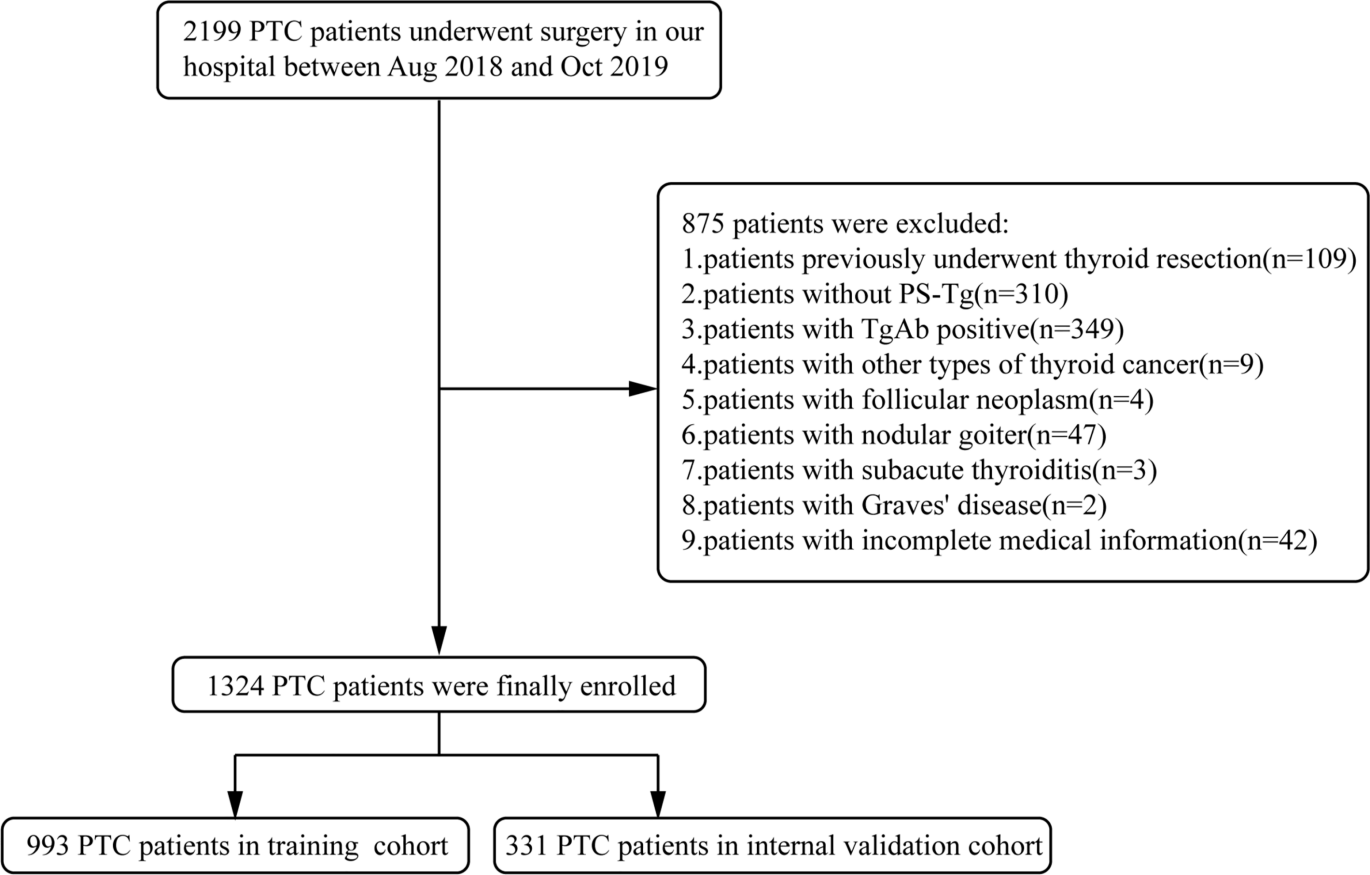
Enrollment flowchart of participants for model development and validation. PTC, papillary thyroid carcinoma; PS-Tg, preoperative serum thyroglobulin.

The study was approved by the First Affiliated Hospital of Zhengzhou University Ethics Review Committee. The data of PTC patients who had a surgery at the First Affiliated Hospital of Zhengzhou University from August 2018 to October 2019 were collected. Inclusion criteria were as follows: (1) patients with pathology-confirmed PTC; (2) patients with preoperative serum thyroglobulin within 1 week; and (3) patients with postoperative follow-up of at least 2 years. Additionally, exclusion criteria were as follows: (1) patients previously underwent thyroid resection; (2) patients with other type of thyroid cancer; (3) patients with TgAb positive; (4) patients with other thyroid diseases such as follicular neoplasm, nodular goiter (≥2 cm), subacute thyroiditis, Graves’ disease, etc.; and (5) patients with incomplete medical information. The selection process is summarized in **Figure 1**. After the establishment of the nomogram model, an external validation cohort was assembled which consisted of 631 PTC patients who underwent surgery in the same hospital from March to November 2021 with the uniform exclusion criteria.

### Background information

Surgical information is as follows: based on preoperative assessment, lobectomy (LT) had been performed for patients that fulfilled the following criteria: (1) maximum tumor diameter <4 cm; (2) without ETE; (3) without bilateral or multifocal tumors; and (4) without clinical evidence of lymph node metastasis or distant metastasis. Otherwise, total thyroidectomy (TT) was chosen. Ipsilateral central neck lymph node dissection (CLND) was routinely performed, and bilateral CLND was only performed in patients with clinical evidence of contralateral CLNM. The extent of CLND was the hyoid bone superiorly, the suprasternal fossa inferiorly, and the carotid sheaths laterally, encompassing the prelaryngeal, pretracheal, and ipsilateral paratracheal lymph nodes. Lateral neck lymph node dissection (LLND) from levels II to V was only performed in patients with clinically suspected or definite LLNM before or during surgery. The extent of LLND was digastric muscles superiorly, clavicle inferiorly, carotid sheaths medially, and trapezius muscles laterally.

Lymph node metastasis was considered negative if no lymph nodes were examined in the perioperative period and no regional recurrence postoperative reported within 2 years. The pathological approach was as follows: all of the acquired surgical specimens were examined by at least two board-certified pathologists from the department of pathology of the First Affiliated Hospital of Zhengzhou University. Pathological features included the pathological type of the tumor, the type of surrounding thyroid tissue, tumor size, ETE (including capsular invasion), multifocality, and lymph node metastasis (region and number).

Thyroid function tests showed the following: TSH (reference: 0.34–5.60 mIU/ml), Tg (reference: 3.5–77.00 ng/ml), TgAb (>115.00 IU/ml means positive), TPOAb (>34.00 IU/ml means positive).

### Statistical analysis

The PTC patients (n = 1,324) were randomly divided into the training group (n = 993) and the validation group (n = 331). To describe the characteristics of the two groups, descriptive statistics of all variables, including medians and proportions, were used.

The categorical data were expressed as percentage. In addition, not satisfying the normal distribution, the continuous variables were reported as medians (quartile 1, quartile 3). To compare the baseline clinical characteristics of the two groups, Pearson’s chi-square test and Wilcoxon test were applied. P values were derived from two-tailed tests. Univariate and multivariate logistic regression analyses were performed to identify the independent risk factors for CLNM/LLNM in PTC patients. Consequently, to make it convenient for clinical application, variables with a P < 0.05 in the multivariate analysis were used to develop the risk prediction model and to construct the nomogram.

To evaluate the discriminative power and consensus of the established prediction model, a receiver operating characteristic (ROC) curve was drawn and the area under the receiver operating characteristic curve (AUC) was used. While the performance of the nomogram was further assessed by the calibration curve, which plotted the predicted probability of the nomogram against the observed probability, the fitting degree of the model was assessed by the Akaike information criterion (AIC). Furthermore, to determine the clinical utility of the prediction model, decision curve analysis was constructed by quantifying the net benefits at different threshold probabilities. Finally, to assess the predictive ability of the model, we performed validation processes on 631 potentially relevant cases as external validation.

All of the processes of statistical analysis were completed by R software, version 4.1.1. P value <0.05 was considered statistically significant. Model validation and evaluation processes were independently performed on the training and validation cohorts, respectively. The work has been reported to be compliant with the STROCSS criteria (21).

## Results

### Baseline clinical characteristics of patients with PTC

The baseline characteristics of the training group (n = 993) and the validation group (n = 331) are shown in **Table 1**. The training and internal validation groups had no significant differences with regard to such clinicopathological characteristics as age, sex, maximum tumor size, multifocality, CLNM, LLNM, TPOAb level, BRAF^V600E^ mutation, ETE, TSH level, and PS-Tg level.

**Table 1:** Baseline clinical characteristics of PTC patients.

Characteristics	Training cohort	Internal validation cohort	*P* value
	n = 993	n = 331	
Sex			
Male	250 (25.2%)	88 (26.6%)	
Female	743 (74.8%)	243 (73.4%)	0.610
Age (Y)			
≥45	183 (18.4%)	59 (17.8%)	
<45	810 (81.6%)	272 (82.2%)	0.805
TSH (mIU/mL)			
Median (quartile 1, quartile 3)	2.51 (1.68, 3.68)	2.75 (1.805, 3.79)	0.123
Tg (mg/L)			
Median (quartile 1, quartile 3)	15.74 (8.46, 32.60)	16.970 (8.775, 31.900)	0.671
TPOAb			
Negative	921 (92.7%)	303 (91.5%)	
Positive	72 (7.3%)	28 (8.5%)	0.471
ETE			
Negative	878 (88.4%)	286 (86.4%)	
Positive	115 (11.6%)	45 (13.6%)	0.330
Maximum tumor size (cm)			
≤1	749 (56.4%)	247 (58.4%)	
>1	244 (28.8%)	84 (28.6%)	0.769
Multifocality			
Negative	753 (75.8%)	242 (73.1%)	
Positive	240 (24.2%)	89 (26.9%)	0.322
BRAF ^V600E^ mutation			
Negative	129 (13.0%)	46 (13.9%)	
Positive	674 (67.9%)	223 (67.4%)	
Unknown	190 (19.1%)	62 (18.4%)	0.912
CLNM			
Negative	651 (65.6%)	212 (64.0%)	
Positive	342 (34.4%)	119 (36.0%)	0.618
LLNM			
Negative	889 (89.5%)	296 (89.4%)	
Positive	104 (10.5%)	35 (10.6%)	0.959

PTC, papillary thyroid carcinoma; TSH, thyroid stimulating hormone; Tg, thyroglobulin; ETE, extrathyroidal extension; CLNM, central lymph node metastasis; LLNM, lateral lymph node metastasis.

### Clinical factors associated with CLNM and LLNM in the training group

**Figure 2:**
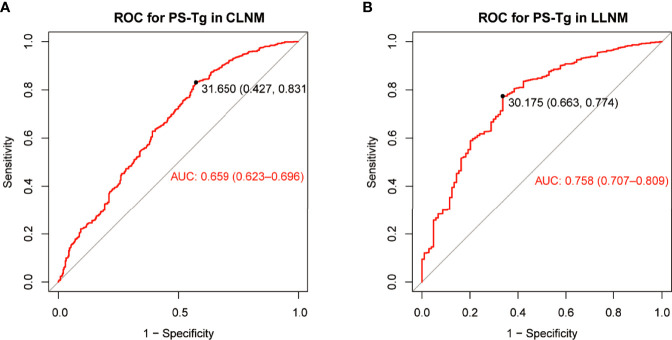
The ROC curve and optimal cutoff value of PS-Tg for CLNM diagnosis **(A)** and LLNM diagnosis **(B)**. PS-Tg, preoperative serum thyroglobulin; CLNM, central lymph node metastasis; LLNM, lateral lymph node metastasis.

The ROC curve of PS-Tg for CLNM diagnosis showed the AUC to be 0.659 (95% CI: 0.623–0.696) (**Figure 2A**) and the optimal cutoff value to be 31.650 ng/ml (the sensitivity was 0.831 and the specificity was 0.427). Then, the Tg level was dichotomized based on the optimal cutoff value and analyzed as categorical variables. Using univariate logistic regression, CLNM was shown to be significantly associated with sex, age, PS-Tg level ≥31.650, ETE, maximum tumor size, and multifocality (all P < 0.05) (**Table 2**). Subsequently, multivariate logistic regression modeling was conducted to screen for significant variables associated with CLNM. As shown in **Table 2**, the results revealed that male (OR: 2.10, 95% CI: 1.49–2.97, P < 0.001), age ≥45 (OR: 0.44, 95% CI: 0.32–0.60, P < 0.001), PS-Tg level ≥31.650 (OR: 1.88, 95% CI: 1.32–2.68, P < 0.001), ETE negative (OR: 0.57, 95% CI: 0.36–0.92, P = 0.02), maximum tumor size >1 cm (OR: 6.47, 95% CI: 4.51–9.37, P < 0.001), and multifocality positive (OR: 2.85, 95% CI: 2.01–4.04, P < 0.001) remained independent predictive variables for CLNM.

**Table 2:** Univariate and multivariate analyses of factors associated with CLNM in the training cohort.

Characteristics	Univariate analysis (CLNM)	*P* value	Multivariate analysis (CLNM)	*P* value
	OR (95% CI)		OR (95% CI)	
Sex				
Male	2.12 (1.58–2.84)	<0.001	2.10 (1.49–2.97)	<0.001
Female				
Age (Y)				
≥45	0.52 (0.4–0.68)	<0.001	0.44 (0.32–0.60)	<0.001
<45				
TSH (mIU/mL)	0.99 (0.95–1.04)	0.83	–	–
PS-Tg (mg/L)				
≥31.65 mg/L	3.66 (2.72–4.93)	<0.001	1.88 (1.32–2.68)	<0.001
<31.65 mg/L				
TPOAb				
Negative	1.4 (0.82–2.38)	0.22	1.09 (0.49–2.45)	0.83
Positive				
ETE				
Negative	0.34 (0.23–0.51)	<0.001	0.57 (0.36–0.92)	0.02
Positive				
Maximum tumor size (cm)				
≤1				
>1	8.6 (6.2–11.91)	<0.001	6.47 (4.51-9.37)	<0.001
Multifocality				
Negative				
Positive	2.81 (2.09–3.79)	<0.001	2.85 (2.01-4.04)	<0.001
BRAF^V600E^ mutation				
Negative				
Positive	1.43 (0.95–2.16)	0.09	–	–
Unknown	1.12 (0.69–1.83)	0.65	–	–

CLNM, central lymph node metastasis; LLNM, lateral lymph node metastasis; TSH, thyroid stimulating hormone; PS-Tg, preoperative serum thyroglobulin; ETE, extrathyroidal extension.

The ROC curve of PS-Tg for LLNM diagnosis indicated that the AUC was 0.758 (95% CI: 0.707–0.809) (**Figure 2B**), and the optimal cutoff value was 30.175 ng/ml (with the sensitivity and specificity of 0.774 and 0.663, respectively). The univariate analysis revealed a significant relationship between LLNM and such factors as sex, age, PS-Tg level ≥30.175, ETE, maximum tumor size, multifocality, and CLNM status (all P < 0.05) (**Table 3**). Additionally, after implementing the multivariable logistic regression analysis, five significant variables were found to be associated with LLNM. As is seen in **Table 3**, the variables included PS-Tg level ≥30.175 (OR: 2.72, 95% CI: 1.63–4.56, P < 0.001), ETE negative (OR: 0.35, 95% CI: 0.19–0.62, P < 0.001), maximum tumor size >1 cm (OR: 3.01, 95% CI: 1.76–5.24, P < 0.001), multifocality positive (OR: 2.11, 95% CI: 1.26–3.55, P < 0.01), and CLNM negative (OR: 0.08, 95% CI: 0.03–0.16, P<0.001).

**Table 3:** Univariate and multivariate analyses of factors associated with LLNM in the training cohort.

Characteristics		Univariate analysis (LLNM)	*P* value	Multivariate analysis (LLNM)	*P* value
		OR (95% CI)		OR (95% CI)	
Sex					
Male		1.75 (1.14–2.69)	0.01	1.16 (0.68–1.96)	0.59
Female					
Age (Y)					
≥45		0.60 (0.40–0.90)	0.01	0.69 (0.41–1.15)	0.15
<45					
TSH (mIU/mL)		0.96 (0.86–1.07)	0.45	–	–
PS-Tg (mg/L)					
≥30.175 mg/L		6.75 (4.36–10.44)	<0.001	2.72 (1.63–4.56)	<0.001
<30.175 mg/L					
TPOAb					
Negative		1.09 (0.49–2.45)	0.83	–	–
Positive					
ETE					
Negative		0.18 (0.12–0.29)	<0.001	0.35 (0.19–0.62)	<0.001
Positive					
Maximum tumor size (cm)					
≤1					
>1		11.65 (7.32–18.54)	<0.001	3.01 (1.76–5.24)	<0.001
Multifocality					
Negative					
Positive		3.26 (2.15–4.94)	<0.001	2.11 (1.26–3.55)	<0.01
BRAF^V600E^ mutation					
Negative					
Positive		1.64 (0.8–3.38)	0.18	–	–
Unknown		1.66 (0.73–3.74)	0.22	–	–
CLNM					
Negative		0.03 (0.02–0.07)	<0.001	0.08 (0.03–0.16)	<0.001
Positive					

CLNM, central lymph node metastasis; LLNM, lateral lymph node metastasis; TSH, thyroid stimulating hormone; PS-Tg, preoperative serum thyroglobulin; ETE, extrathyroidal extension.

### Development of the nomogram for predicting CLNM and LLNM in PTC patients

**Figure 3:**
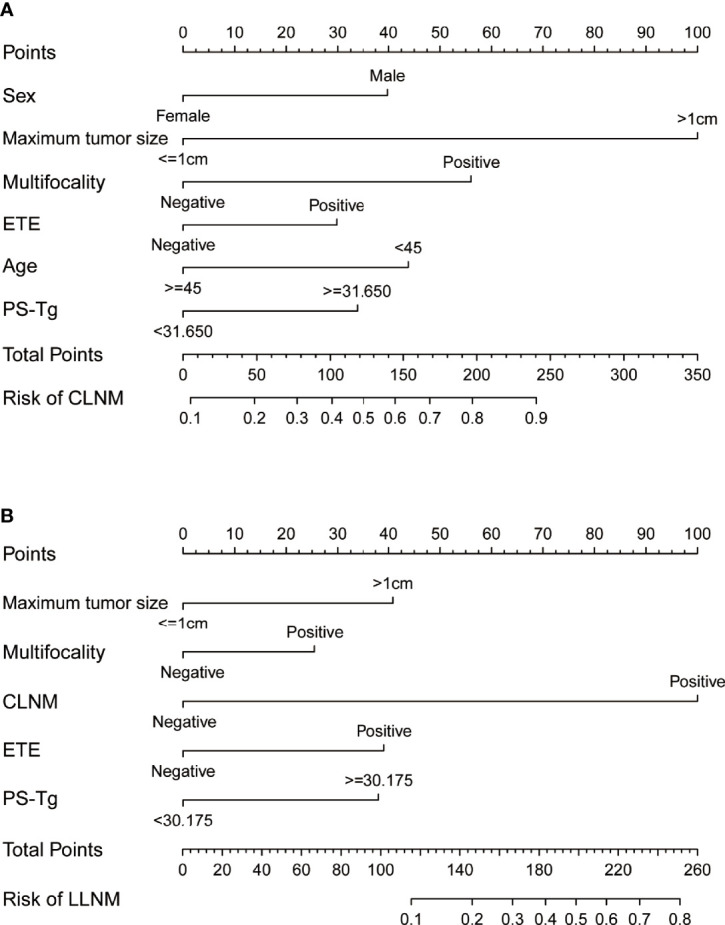
The nomograms indicate the risk of CLNM **(A)** and LLNM **(B)** based on clinical factors. CLNM, central lymph node metastasis; LLNM, lateral lymph node metastasis; TSH, thyroid stimulating hormone; PS-Tg, preoperative serum thyroglobulin; ETE, extrathyroidal extension.

For facilitating the estimation of the metastasis risk of CLNM and LLNM for individuals with PTC as well as making the model convenient to use, all the risk factors shown to be statistically significant in the logistic regression model were included in the nomogram (**Figures 3A, B**). Based on the regression coefficient of CLNM or LLNM, each variable was proportionally assigned a point on a scale from 0 to 100. The nomogram confirmed the maximum tumor size >1 cm as the largest contributor to CLNM, while CLNM positive as the largest contributor to LLNM. The value of each variable was represented as a score by drawing a straight line upward from the corresponding value to the “Points” line. By adding the total score and positioning it on the scale of the total points, the corresponding probability of CLNM or LLNM in each person was determined.

### Validation of the prediction nomogram

**Figure 4:**
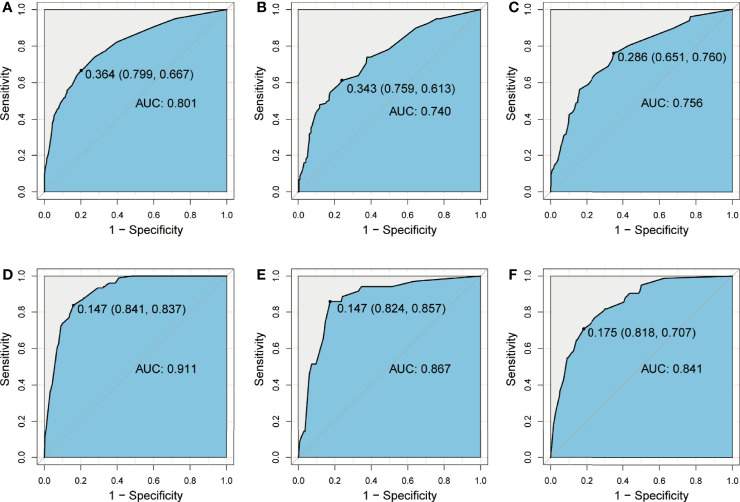
ROC curve showing prediction effective of the nomogram model for CLNM in the training group **(A)**, the internal validation group **(B),** and the external validation group **(C)**, as well as nomogram model for LLNM in the training group **(D)**, the internal validation group **(E),** and the external validation group **(F)**. CLNM, central lymph node metastasis; LLNM, lateral lymph node metastasis.

The model, then, was used to perform ROC analysis on the training and validation groups. The areas under the curve (AUCs), referred to as C-statistic, were considered to be an indicator in evaluating the effectiveness of the model. With an AUC of 0.801 (cutoff value: 0.364, sensitivity: 0.667, specificity: 0.799; **Figure 4A**), the CLNM prediction model demonstrated a high efficiency for the training group. The effectiveness in the internal validation and external validation groups was subsequently verified with the superior efficiency of an AUC of 0.740 (cutoff value: 0.343, sensitivity: 0.613, specificity: 0.759; **Figure 4B**) and 0.756 (cutoff value: 0.286, sensitivity: 0.760, specificity: 0.651; **Figure 4C**), respectively. The high value of AUC was an indication of the great ability of the model in CLNM prediction. Likewise, the prediction model for LLNM showed an AUC of 0.911 (cutoff value: 0.147, sensitivity: 0.837, specificity: 0.841; **Figure 4D**) in the training group. Similarly, the effectiveness of the model was verified in the internal validation and external validation groups, with a superior efficiency of AUC of 0.867 (cutoff value: 0.147, sensitivity: 0.857, specificity: 0.824; **Figure 4E**) and 0.841 (cutoff value: 0.175, sensitivity: 0.707, specificity: 0.818; **Figure 4F**) were obtained, respectively.

**Figure 5:**
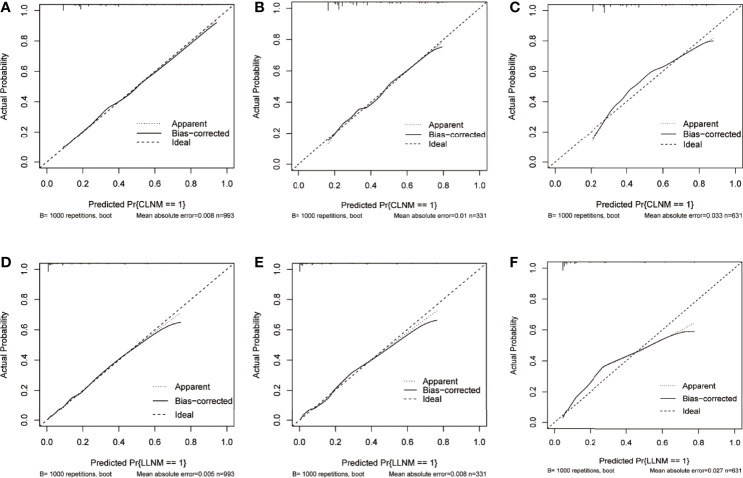
Calibration curve of the prediction nomogram for CLNM in the training group **(A)**, the internal validation group **(B),** and the external validation group **(C)**, as well as nomogram model for LLNM in the training group **(D)**, the internal validation group **(E),** and the external validation group **(F)**. The x-axis represents the predicted probability of CLNM or LLNM, while the y-axis stands for the actual diagnosed probability of CLNM or LLNM. The diagonal dashed line represents an ideal prediction model. The solid line shows the performance of the nomogram models, of which a closer fit to the diagonal dashed line indicates better prediction ability. CLNM, central lymph node metastasis; LLNM, lateral lymph node metastasis.

Furthermore, a similar bootstrap resampling procedure was used to conduct the internal and external calibration plots for the established model. For CLNM, the calibration curves demonstrated a good agreement between prediction and observation in the training group (mean absolute error = 0.008), the internal validation group (mean absolute error = 0.01), and the external validation group (mean absolute error = 0.033, **Figures 5A–C**). A good correspondence was also found between the predicted and observed metastasis risks of LLNM; only minor discrepancies were observed in the training group (mean absolute error = 0.005), the internal validation group (mean absolute error = 0.0058), and the external validation group (mean absolute error = 0.027) (**Figures 5D–F**).

### Decision curve showed it would add more net benefits for clinical decision

**Figure 6:**
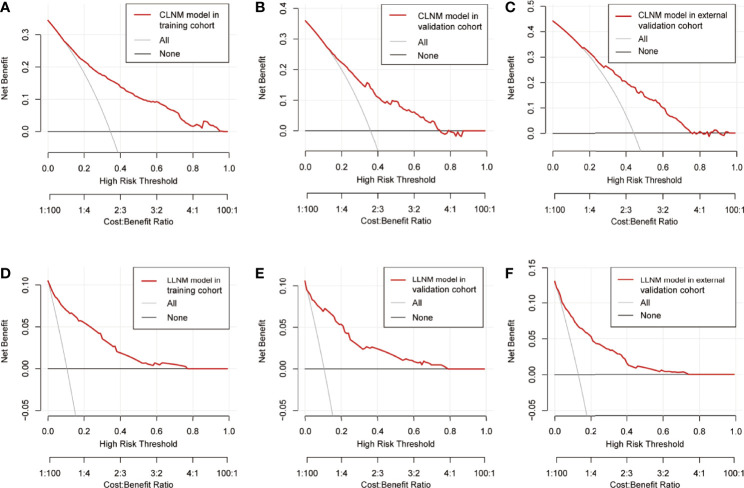
Decision curve of the prediction nomogram for CLNM in the training group **(A)**, the internal validation group **(B),** and the external validation group **(C)**, as well as nomogram model for LLNM in the training group **(D)**, the internal validation group **(E),** and the external validation group **(F)**. The red line represents the nomogram model. The gray line represents the assumption that all patients are CLNM/LLNM positive, while the horizontal black line represents that all patients are CLNM/LLNM negative. CLNM, central lymph node metastasis; LLNM, lateral lymph node metastasis.

The result of decision curve analysis of the nomogram in detecting CLNM/LLM for PTC patients is presented in **Figure 6**. The decision curve proved the efficacy of the nomogram in rather large ranges of threshold probability for each group. The nomogram model for CLNM could be more effective than the all-treated or non-treated strategy when the threshold probability ranged from 0.1 to approximately 0.8 in three cohorts, and the prediction of LLNM could benefit more with a threshold probability ranging from 0 to 0.7.

## Discussion

Concerning the favorable prognosis of PTC, since an unnecessary surgical region increases the risk of such complications as recurrent laryngeal nerve injury, permanent hypoparathyroidism, chyle leakage, sympathetic nerve injury, accessory nerve injury, neuropathic pain of cervical plexus, shoulder weakness, etc. (21–24), the region of surgical resection is also increasingly inclined to be more individualized and conservative. According to ATA guidelines (2015 version) (25), lobectomy is feasible with the absence of LNM and ETE in papillary thyroid microcarcinoma (PTMC). Furthermore, a considerable number of surgeons recommend lobectomy even for tumors with 1- to 4-cm diameters when LNM and ETE are absent. Prophylactic lymph node dissection is still considered a controversial method in PTC patients with no evidence of preoperative LNM. There is literature supporting prophylactic CLND, which shows that prophylactic CLND reduced the risk of postoperative recurrence and secondary surgery (26). Opposing this view, other studies provide evidence that prophylactic CLND had no effect on the survival rate of patients but instead increased postoperative complications and reduced the quality of life (27). A study by Calò et al. found that TT in combination with CLND, especially bilateral CLND, would significantly increase the detection rates of unexpected lymph node metastasis in cN0 patients. Unfortunately, this surgery treatment still made no significant difference in the rates of postoperative recurrence and led to more postoperative complications (28). The scope of the surgery is largely determined by the evaluation of preoperative lymph nodes, which is also closely associated with postoperative recurrence and prognosis. This requires a more accurate evaluation of the preoperative LNM on the side of the surgeons. In line with this, the present study aimed at developing a nomogram model in predicting LNM risk and, accordingly, guiding clinicians in surgical decision-making.

In this study, 1,324 PTC patients with initial thyroid surgery were examined. Based on PS-Tg, TSH, and other clinical characteristics, risk factors in promoting cervical LNM were studied. Moreover, to help the assessment of cervical LNM, nomogram models were established. The rate of LNM was 35.7% (CLNM 34.8% and LLNM 10.5%) in this study, which was comparable to the findings of previous studies (29–31). While sex, age, maximum tumor size, multifocality, and ETE were found as independent risk factors of CLNM through univariate and multivariate analyses, maximum tumor size, multifocality, ETE, and CLNM were found to be the independent risk factors for LLNM. The findings largely correspond to those of the previous studies (32–35). Currently, the association between BRAF ^V600E^ mutation and LNM remains disputable. More studies support a positive correlation between BRAF^V600E^ mutation and LNM (36, 37). In our study, the finding showed no relationship between the variables, which was consistent with those obtained in several others studies (33, 38).

Interestingly, we found that PS-Tg was significantly increased in the cervical LNM group. Tg is synthesized and secreted by thyroid follicular epithelial cells, including normal thyroid follicular epithelial cells and thyroid follicular epithelial cells of DTC foci and metastatic lymph nodes (16). This suggests that greater tumor burden implies greater functional follicular epithelial cells, which potentially leads to higher Tg. Besides, it is also possible that the tumor destroyed follicular structures, which caused the release of Tg into the peripheral blood. Accordingly, other diseases causing Tg abnormity (e.g., follicular tumors, nodular goiter, Graves disease, subacute thyroiditis) (16, 39, 40) as well as factors interfering with the measurement of PS-Tg (e.g., TgAb positive) (41)were excluded in our study.

The ROC curves provided us with the optimal PS-Tg cutoff for predicting cervical LNM (PS-Tg = 31.650 ng/ml for CLNM and PS-Tg = 30.175 ng/ml for LLNM). Furthermore, the multivariate analysis showed that PS-Tg ≥31.650 ng/ml and PS-Tg ≥30.175 ng/ml were independent risk factors for CLNM and LLNM, respectively. Zhou et al. (42) found similar results. Kim et al. (17, 18) also observed that increased PS-Tg was associated with tumor burden (including LNM and distant metastasis), which corroborates the findings of our study. However, in one study, no significant relationship was found between PS-Tg and the prediction of PTC lymph node metastasis (43). Nevertheless, this can be justified by the inclusion of only 234 patients with thyroid cancer in that study (follicular thyroid carcinoma was not excluded). Moreover, diseases causing Tg changing such as those mentioned above were not excluded from that study.

The ROC curve of PS-Tg for the diagnosis of CLNM (AUC: 0.659, sensitivity: 0.831, specificity: 0.427), and LLNM (AUC: 0.758, sensitivity: 0.774, specificity: 0.663) showed that the independent predictive power of PS-Tg was still finite. This may imply that PS-Tg could have a more independent predictive value only when the tumor burden reached a certain degree. In other words, the greater the tumor burden, the higher the predictive value of PS-Tg for LNM. Therefore, although the PS-Tg cutoff value of predicting CLNM and LLNM was close, the AUC and specificity of the latter were more valuable than the former. As a result, prediction of LNM based on Tg alone is still insufficient and is required to be combined with other relevant clinical features.

Through review of the literature on developing prediction models of LNM in PTC patients, we found that PS-Tg was only taken by Zhou et al. (42) as a factor of predicting model for CLNM. Nevertheless, the best cutoff value of PS-Tg was not examined in their study, due to a low prediction score of PS-Tg in the nomogram model. Moreover, numerous studies focused on ultrasound characteristics of tumor to predict LNM (30, 44). However, the ultrasound characteristics largely rely on the doctors’ experience, which leads to subjective bias, easily affecting the objectivity of the obtained results. Liu et al. (33) developed a nomogram model of LLNM based on BRAF^V600E^ protein status. The model, however, yielded a poor C-index of 0.714. Nonetheless, BRAF^V600E^ protein status was preoperatively unavailable in most cases. In the nomogram models established by Kim et al. (10), Wang et al. (45), and Feng et al. (29), the clinical characteristics of a large number of patients were investigated. However, all three models exhibited unsatisfying precision with C-indices of 0.721, 0.715, and 0.733, respectively.

Combined with previous findings and based on the risk factors affecting LNM in our analysis, we selected factors with higher predictive potentials to include in the nomogram model. Finally, being male, age, maximum tumor size, multifocality, ETE, and PS-Tg ≥31.65 mg/l showed high predictive potential in both univariate and multivariate analyses. Hence, they were included in the nomogram model for CLNM. Similarly, maximum tumor size, multifocality, ETE, CLNM, and PS-Tg ≥30.175 mg/l were included in the nomogram model for the prediction of LLNM. Surprisingly, both nomogram models exhibited high precisions, with a C-index of 0.801 in CLNM and 0.911 in LLNM predicting models. In addition, the two nomograms were further successfully evaluated by the internal and external validation cohorts. The calibration curves in both the training and validation cohorts revealed a favorable agreement between the ideal and bias-corrected lines by the nomogram developed in the present study. Moreover, the decision curve graphically showed the clinical benefits of the model. Using our nomogram, clinicians can assess the probability of preoperative LNM effectively, which can assist them in surgical decision-making.

Nonetheless, there are still a number of limitations in this research. Firstly, being a single-center retrospective study, there are inherent biases. Thus, more multicenter prospective studies are needed in the future. Secondly, although such non-PTC-related factors as TgAb positive, follicular neoplasm, nodular goiter, subacute thyroiditis, and Graves’ disease could clearly affect or interfere PS-Tg levels, the results might still be influenced by some undiscovered non-PTC-related factors. As a consequence, since the non-PTC-related factors were eliminated, the application scope of the proposed model is limited. Finally, only six predictive indicators were incorporated in our nomogram model, which suggests the integration of more influencing factors in future to further improve the predictive ability of the model.

In summary, we found the PS-Tg level to be an independent risk factor of LNM.PS-Tg can assist the prediction of LNM. Moreover, to predict CLNM, a nomogram model was established based on PS-Tg, male, age, max tumor diameter, multifocality, and ETE. Furthermore, a nomogram model was also established based on PS-Tg, max tumor diameter, multifocality, ETE, and CLNM to predict LLNM, the use of which can benefit clinicians in assessing the probability of preoperative LNM and making surgical decisions.

## Data availability statement

The original contributions presented in the study are included in the article/supplementary material. Further inquiries can be directed to the corresponding author.

## Ethics statement

The studies involving human participants were reviewed and approved by The First Affiliated Hospital of Zhengzhou University Ethics Review Committee. Written informed consent for participation was not required for this study in accordance with the national legislation and the institutional requirements.

## Author contributions

QC and JZ conceptualized the study and wrote the original draft. QC, YW, HL, and XD performed the data collection and validation. JZ and DZ analyzed the data and completed the figures. DY provided the study material and made critical revisions to the manuscript. All authors contributed to the article and approved the submitted version.

## Funding

This work was supported by the Medical Science and Technology Project of Henan Province (grant number: LHGJ20190012), General Project of Natural Science Foundation of Henan Province (grant number: 222300420568), Key Medical Science and Technology Project of Henan Province (grant number: SBGJ202101014), Major Scientific Research Projects of Traditional Chinese Medicine in Henan Province (grant number: 20-21ZYZD14), and Cultivation of Young and Middle-aged Health Science and Technology Innovation Leading Talents in Henan Province (grant number: YXKC2020015).

## Conflict of interest

The authors declare that the research was conducted in the absence of any commercial or financial relationships that could be construed as a potential conflict of interest.

## Publisher’s note

All claims expressed in this article are solely those of the authors and do not necessarily represent those of their affiliated organizations, or those of the publisher, the editors and the reviewers. Any product that may be evaluated in this article, or claim that may be made by its manufacturer, is not guaranteed or endorsed by the publisher.
